# Supplementary figures and images for: Transcriptome Analysis of the Effects of Fasting Caecotrophy on Hepatic Lipid Metabolism in New Zealand Rabbits
Source: Animals (Basel). 2019 Sep 3;9(9):648. doi: 10.3390/ani9090648 (PMC6769842; doi:10.3390/ani9090648)

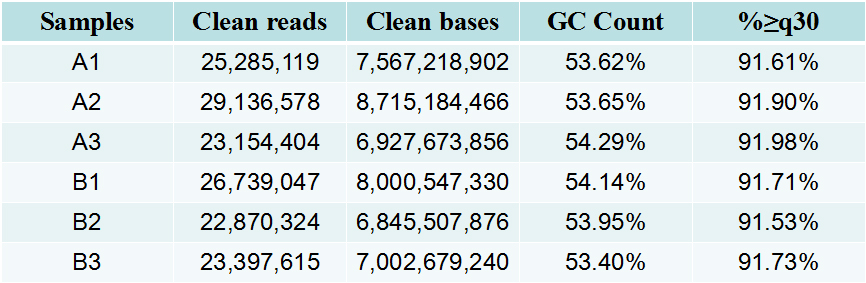

Supplement: Supplementary file 1 [file animals-09-00648-s001.zip › supporting information/S1 map.tif]

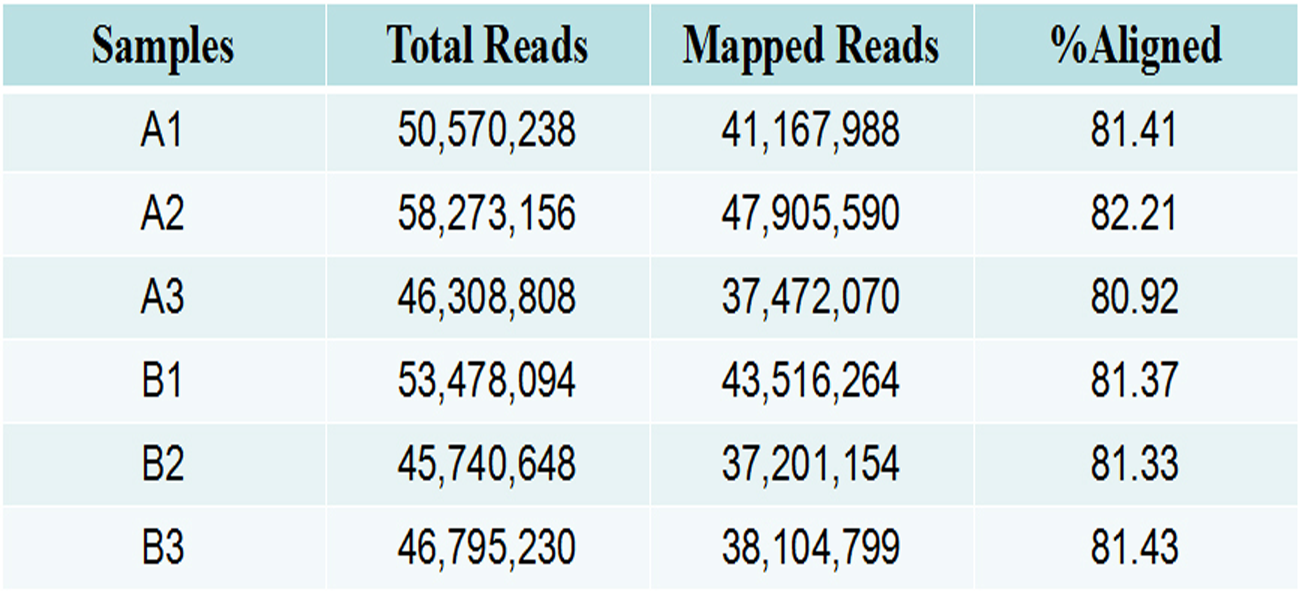

Supplement: Supplementary file 1 [file animals-09-00648-s001.zip › supporting information/S2 map.tif]
